# Effects of clinical pathway implementation on antibiotic prescriptions for pediatric community-acquired pneumonia

**DOI:** 10.1371/journal.pone.0193581

**Published:** 2018-02-28

**Authors:** Daniele Donà, Silvia Zingarella, Andrea Gastaldi, Rebecca Lundin, Giorgio Perilongo, Anna Chiara Frigo, Rana F. Hamdy, Theoklis Zaoutis, Liviana Da Dalt, Carlo Giaquinto

**Affiliations:** 1 Division of Infectious Diseases and the Center for Pediatric Clinical Effectiveness, Children’s Hospital of Philadelphia, Philadelphia, Pennsylvania, United States of America; 2 Division of Pediatric Infectious Diseases, Department for Woman and Child Health, University of Padua, Padua, Italy; 3 PENTA Foundation, Padua, Italy; 4 Pediatric Emergency Department, Department for Woman and Child Health, University of Padua, Padua, Italy; 5 Department for Woman and Child Health, University of Padua, Padua, Italy; 6 Biostatistics, Epidemiology and Public Health Unit, Department of Cardiac, Thoracic and Vascular Sciences, University of Padua, Padua, Italy; 7 Department of Pediatrics, Children's National Health System, Washington DC, United States of America; Mahidol-Oxford Tropical Medicine Research Unit, THAILAND

## Abstract

**Background:**

Italian pediatric antimicrobial prescription rates are among the highest in Europe. As a first step in an Antimicrobial Stewardship Program, we implemented a Clinical Pathway (CP) for Community Acquired Pneumonia with the aim of decreasing overall prescription of antibiotics, especially broad-spectrum.

**Materials and methods:**

The CP was implemented on 10/01/2015. We collected antibiotic prescribing and outcomes data from children aged 3 months-15 years diagnosed with CAP from 10/15/2014 to 04/15/2015 (pre-intervention period) and from 10/15/2015 to 04/15/2016 (post-intervention period). We assessed antibiotic prescription differences pre- and post-CP, including rates, breadth of spectrum, and duration of therapy. We also compared length of hospital stay for inpatients and treatment failure for inpatients and outpatients. Chi-square and Fisher’s exact test were used to compare categorical variables and Wilcoxon rank sum test was used to compare quantitative outcomes.

**Results:**

120 pre- and 86 post-intervention clinic visits were identified with a diagnosis of CAP. In outpatients, we observed a decrease in broad-spectrum regimens (50% pre-CP vs. 26.8% post-CP, p = 0.02), in particular macrolides, and an increase in narrow-spectrum (amoxicillin) post-CP. Post-CP children received fewer antibiotic courses (median DOT from 10 pre-CP to 8 post-CP, p<0.0001) for fewer days (median LOT from 10 pre-CP to 8 post-CP, p<0.0001) than their pre-CP counterparts. Physicians prescribed narrow-spectrum monotherapy more frequently than broad-spectrum combination therapy (DOT/LOT ratio 1.157 pre-CP vs. 1.065 post-CP). No difference in treatment failure was reported before and after implementation (2.3% pre-CP vs. 11.8% post-CP, p = 0.29). Among inpatients we also noted a decrease in broad-spectrum regimens (100% pre-CP vs. 66.7% post-CP, p = 0.02) and the introduction of narrow-spectrum regimens (0% pre-CP vs. 33.3% post-CP, p = 0.02) post-CP. Hospitalized patients received fewer antibiotic courses post-CP (median DOT from 18.5 pre-CP to 10 post-CP, p = 0.004), while there was no statistical difference in length of therapy (median LOT from 11 pre-CP to 10 post-CP, p = 0.06). Days of broad spectrum therapy were notably lower post-CP (median bsDOT from 17 pre-CP to 4.5 post-CP, p <0.0001). No difference in treatment failure was reported before and after CP implementation (16.7% pre-CP vs. 15.4% post-CP, p = 1).

**Conclusions:**

Introduction of a CP for CAP in a Pediatric Emergency Department led to reduction of broad-spectrum antibiotic prescriptions, of combination therapy and of duration of treatment both for outpatients and inpatients.

## Background

Pneumonia is the single greatest cause of death in children worldwide: 1–4% of the pediatric population is treated every year for community acquired pneumonia (CAP) and 0.1–2% of those children are hospitalized [1–3].

Inpatient healthcare costs associated with CAP are estimated to be more than one billion dollars per year [4].

Inappropriate antibiotic prescribing for CAP has been frequently reported, as many patients receive antibiotics for viral pneumonia or broad-spectrum antibiotics for uncomplicated bacterial pneumonia [5]. The Italian antimicrobial prescription rate is one of highest in the EU (52%) [6], and antibiotic resistance has become a serious health threat with high social costs and severe consequences including prolonged illness, increased length of hospitalization and mortality [6]. Increasing penicillin and macrolide resistance of *Streptococcus pneumoniae* strains pose an important threat to effective treatment [7]. There is also widespread β-lactamase production in *Haemophilus influenzae* and macrolide resistance in *Streptococcus pyogenes* [8].

Thus, it is imperative to reduce improper use of these drugs. Clinical Pathways (CPs) along with educational programs have shown to be a reasonable and feasible first step for Antimicrobial Stewardship Program (ASP) implementation by reducing antibiotic prescriptions in both community and in-hospital settings [9–13].

To date, ASPs have primarily targeted the inpatient setting, and there is a paucity of literature regarding antimicrobial stewardship strategies in the Pediatric Emergency Department (PED), despite the substantial proportion of antibiotics prescribed to children in this setting [14–17]. Since PEDs are uniquely positioned at the interface of inpatient and outpatient settings, PED physicians could have a consistent impact on prescribing trends in both locations.

In the PED setting, challenges include high turnover rates for both patients and practitioners, the need for rapid decision-making, and diagnostic uncertainty in empiric prescription [18].

Since CPs have been effective in reducing antibiotic prescriptions in primary care and in hospital settings, we hypothesized that their implementation in the PED could decrease overall prescription of antibiotics, especially broad-spectrum, for common infectious diseases such as CAP [9–13].

The primary aim of this study was to assess changes in antibiotic prescription before and after CP implementation for CAP in a large Italian PED. Secondary aims were to compare treatment failures before and after CP implementation.

## Materials and methods

### Study design

The study was set at the PED of the Department for Women and Children Health at Padua University Hospital. Our Children’s Hospital provides primary and secondary care for a metropolitan area of 350,000 people (45,000 younger than 15 years) and tertiary care for a regional and extra-regional population, with approximately 26,000 PED visits per year and an overall hospital admission rate from PED of around 7 out of 100 visits.

From the PED, children with moderate-severe CAP (criteria listed in the CP S1 Fig) are usually admitted to the Pediatric Acute Care Unit (PACU), an acute care unit near the emergency department, which shares the same medical staff.

This is a pre-post quasi-experimental study that assesses the changes in antibiotic prescribing for CAP during a 6-month period preceding CP implementation (pre-intervention, from 15 October 2014 to 15 April 2015) and during the six months after CP implementation (post intervention, from 15 October 2015 to 15 April 2016). The decision to analyse the same period in different years was made in order to limit the effects of seasonality.

### Intervention

On 1 October 2015 CPs for the management of CAP were implemented.

The CP is a one-page decision support algorithm designed to assist providers in determining whether an antibiotic should be prescribed, and if so, the optimal agent and duration of therapy.

The CP summarizes international guidelines [1,8] for the diagnosis and treatment of the clinical condition and was developed by the Division of Pediatric Infectious Diseases and Pediatric Emergency Department of Padua in collaboration with the Division of Pediatric Infectious Diseases of the Children’s Hospital of Philadelphia.

Three CP training sessions (two during the first weeks of October and one during the first week of November) were presented to PED and Pediatric Acute Care Unit (PACU) physicians and residents along with an overview of the guidelines, the rationale behind the treatment.

### Study population

All patients aged between 3 months and 15 years with *International Classification of Diseases*, *9th Revision*, *Clinical Modification* (ICD-9-CM) codes 485 and 486 at discharge diagnosis or descriptive diagnosis of CAP were included.

Exclusion criteria were: cystic fibrosis or other chronic pulmonary diseases (except for asthma), immunodeficiency or immunosuppressive therapy, sickle cell disease, tracheostomy, patients at risk for aspiration pneumonia, hospitalization during previous 30 days, concomitant infections, ongoing antibiotic therapy.

Participating patients were divided in two groups:

- **Outpatients**: patients evaluated at the PED and discharged;
- **Inpatients**: patients admitted to the PACU.

### Data source

All patients with a clinical diagnosis of pneumonia (medical progress notes) or documentation of a chest infiltrate (radiology notes) were included. All clinical, demographic, diagnostic and antimicrobial data were manually collected from electronic medical records, using a password protected REDCap® data collection form and stored in the secure server at the University of Padua.

We considered treatment based on amoxicillin or ampicillin alone narrow-spectrum. Broad-spectrum antimicrobials were defined as: β-lactam and β-lactamase inhibitor combinations, second- and third-generation cephalosporins, clindamycin, glycopeptides, fluoroquinolones and macrolides. Therapeutic regimens including at least one broad-spectrum prescription, despite the association with amoxicillin, were considered broad-spectrum. In line with expert consensus CAP guidelines [1], our CP suggested a dosage of amoxicillin of 90 mg/kg/day divided every 8 hours.

Amoxicillin *per os* rather than penicillin G is recommended due to its better gastrointestinal absorption and higher levels in blood and lung parenchyma [1,18].

Privacy was guaranteed in two ways: a unique, study specific survey number was assigned to each patient and no personally identifying data were collected.

To evaluate the effectiveness and safety of the intervention, follow up phone calls to the family were made within 30 days to assess for treatment failure, defined as new admission, prescription of a new antibiotic (instead of or in addition to the previous one) for persistence or relapse of symptoms or for drug side effects (eg. rash, diarrhea) within 30 days after discharge.

Admissions for CAP in the same patient occurring greater than 30 days apart were analysed as separate events.

This study was approved by the Institutional Review Board of Department for Woman and Child Health at the University of Padua.

### Determination of outcomes

#### Primary outcome

The following aspects of antibiotic prescriptions for CAP were assessed every month over the six months before and the six months after CP implementation:

- Narrow-spectrum prescription rate;
- Duration of therapy expressed in Days of therapy (DOT) and Length of Therapy (LOT) [19–21], DOT/LOT ratio, median DOTs of broad-spectrum antibiotics (bsDOTs) and bsDOT/DOT ratio.
- Dosage of the most frequently prescribed antibiotics, expressed in mg/kg/day;
- Length of hospital stay (LOS) for inpatients.

#### Secondary outcome

Thirty-day treatment failures investigated through a phone call, defined as: changes in antibiotic prescription for persistence or worsening of symptoms; treatment changes for antibiotic side effects or new antibiotic prescriptions within 30 days from discharge date for relapse of symptoms and mortality.

### Data analysis

Results are summarized as frequencies and proportions for categorical variables and as median and range for quantitative variables.

Comparisons of categorical and quantitative variables were conducted with chi-square or Fisher’s exact test and Wilcoxon rank sum test respectively, since the data were not normally distributed (Shapiro-Wilk test). Statistical significance was declared for p ≤0.05. Statistical analysis was conducted with SAS 9.2 (SAS Institute, Inc., Cary, NC) for Windows.

## Results

Over the 6-month pre-intervention period, 13,262 children were evaluated in the PED and 12,335 children were seen during the 6-month post-intervention period.

**Fig 1:**
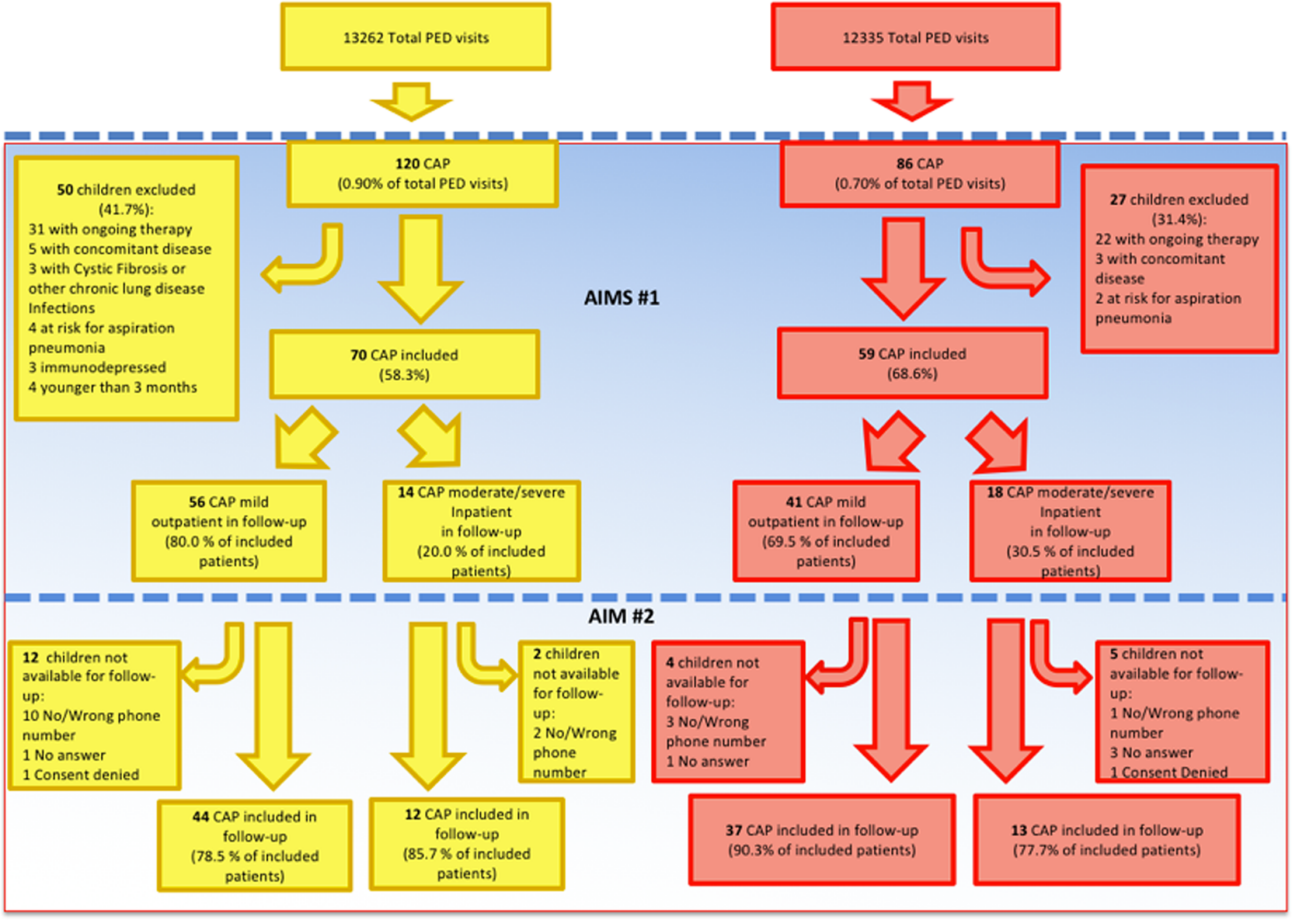
Flowchart of children enrolled during the pre and post-intervention period.

During the pre-intervention period, 120 patients were diagnosed with CAP, accounting for 0.90% (120/13,262) of total PED visits. In the post-intervention period 86/12,335 (0.70%) children were evaluated for CAP. Of these, 70/120 (58.3%) children and 59/86 (68.6%) met the inclusion criteria in the two analysed periods of time (Fig 1).

### Characteristics of the studied population

Variables including sex, age and severity were assessed in pre- and post-CP populations. The two groups were similar with respect to sex (p = 0.76): 50.0% (35/70) females pre-intervention and 54.2% (28/59) post-intervention.

Age was stratified into two age ranges: 3 months-5 years and 5 years-15 years. In both groups, the highest prevalence of CAP was reported in the 3 months-5 years years group with 76.8% (43/56) pre and 92.7% (38/41) post-intervention respectively (p = 0.07). The same analysis was performed also for excluded patients with similar results.

For both the inpatient and outpatient groups, there was no statistically significant difference in frequency or proportion of patients diagnosed with *Mycoplasma pneumoniae* by serology. Proportion of inpatients presenting with hypoxemia and with pleural effusion were similar in the pre- and post-CP groups (Tables 1 and 2).

**Table 1:** Characteristics of outpatients population.

		Pre-Intervention Period		Post-Intervention Period		p value
Included 0utpatients		56 (80.0% of included patients)		41 (69.5% of included patients)		
		N	%	N	%	
**Sex**	m	24	42.9	19	46.3	0.73
	f	32	57.1	22	53.7	
**Age**	3 mo—5 yr	43	76.8	38	92.7	0.07
	5 yr—15 yr	13	23.2	3	7.3	
**Mycoplasma Pneumoniae IgM test**	performed	1	1.8	3	7.3	0.31
	not performed	55	98.2	38	92.7	
**Number of M. Pneumoniae IgM positive test/number of test performed**		1	100	0	0	0.40

**Table 2:** Characteristics of inpatient population.

		Pre-Intervention Period		Post-Intervention Period		p value
Included Inpatients		14 (20.0% of included patients)		18 (30.5% of included patients)		
		N	%	N	%	
**Sex**	m	11	78.6	8	44.4	0.11
	f	3	21.4	10	55.6	
**Age**	3 mo—5 yr	12	85.8	14	77.8	0.9
	5 yr—15 yr	2	14.2	4	22.2	
**Mycoplasma Pneumoniae IgM test**	performed	8	57.1	13	72.2	0.47
	not performed	6	42.9	5	27.8	
**Number of M. pneumoniae IgM positive test/number of test performed**		3	37.5	2	15.4	0.33
**Hypoxia**		8	57.1	11	72.2	1
**Pleural effusion**		4	28.6	3	16.7	0.67
**Chest Drainage**		1	7.1	0	0	0.44

### Antibiotic prescription in outpatients

#### Changes in prevalence of antibiotic prescriptions for CAP

Before implementation 50% of children (28/56) received exclusively amoxicillin, compared with 73.2% (30/41) after CP release. Due to the high prevalence of combination therapy, further analysis on antibiotic prescriptions were performed using Days of Therapy (DOTs) for each patient. The median DOT for the pre-intervention period was 10 (range, 5–26), the median DOT for the post-intervention period was 8 (range, 5–20) (p<0.0001).

**Fig 2:**
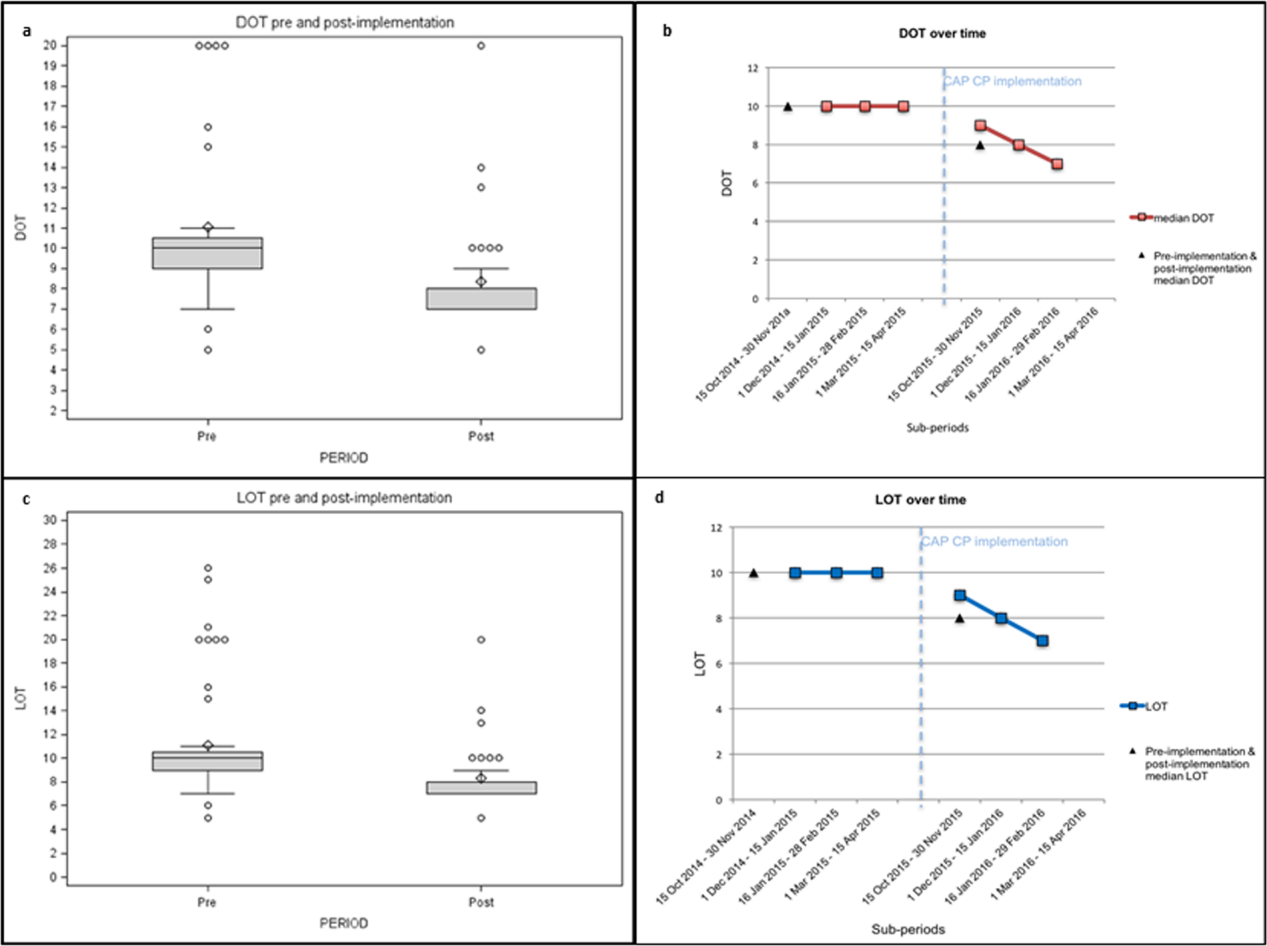
a–b. Median DOT pre and post-implementation for outpatients and DOT over time for outpatients. c. Median LOT pre and post-implementation for outpatients. d. LOT over time for outpatients.

The median DOT was calculated for every sub-period of observation (Fig 2A and 2B). DOTs analysis for each antimicrobial reflected the prescriptions prevalence. Statistically significant increase in use of amoxicillin (54.5% pre-CP vs. 71.1% post-CP, p <0.0001) and decrease in use of macrolides (21.3% pre-CP vs. 6.4% post-CP, p <0.0001) was observed. Cephalosporins and amoxicillin-clavulanate use decreased as well (9.7% and 14.5% pre-CP vs. 8.5% and 14.0% post-CP), but the difference was not statistically significant.

#### Changes in prevalence of broad-spectrum antibiotic prescriptions for CAP

**Fig 3:**
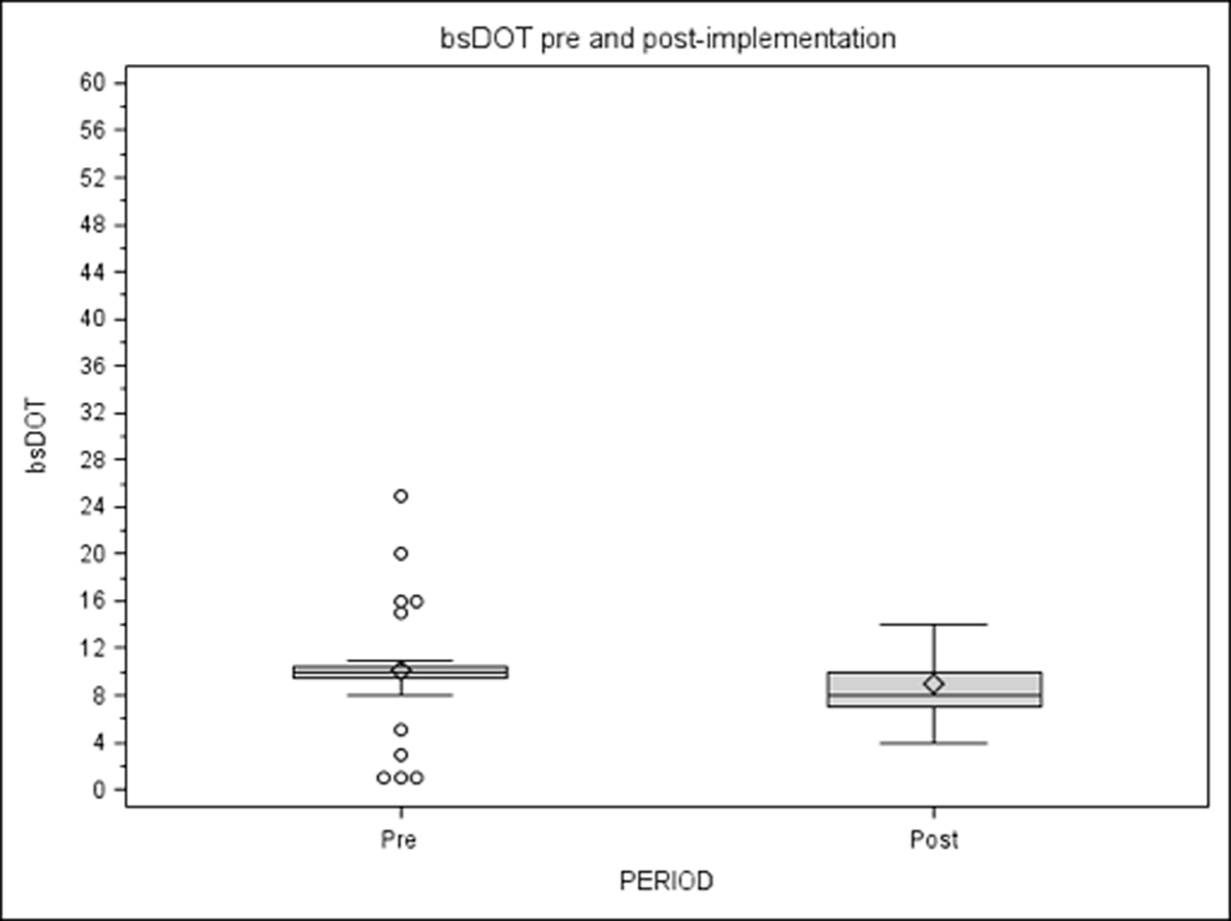
bsDOT/DOT for outpatients.

In the pre-intervention period the median bsDOT was 10 (range 1–25) and in the post-intervention period 8 (range 4–14), with a significant and stable difference in prescribing between pre- and post-intervention groups reported for each sub-period in the time series (Fig 3). As a result, pre-intervention bsDOT/DOT was 0.45, while in post-intervention it was 0.29.

#### Changes in duration of therapy

For treating mild CAP our clinical pathway recommends a 7-day antibiotic therapy.

Pre-intervention median LOT was 10 (range 3–15), while post-intervention median LOT was 8 (range 5–10) (p<0.0001) as recommended in the CP, with a decreasing trend over all sub-periods after implementation.

DOT/LOT ratio indicates use of combination therapy and the length of therapy: pre-intervention DOT/LOT was 1.16, post-intervention 1.07. Specifically, during the related sub-periods, pre-CP DOT/LOT was included between 1.25 and 1.08, post-CP ratio, instead, ranged from 1.19 to 1.04 (Fig 2C and 2D).

#### Changes in dosage for the most commonly prescribed antibiotic for CAP

**Fig 4:**
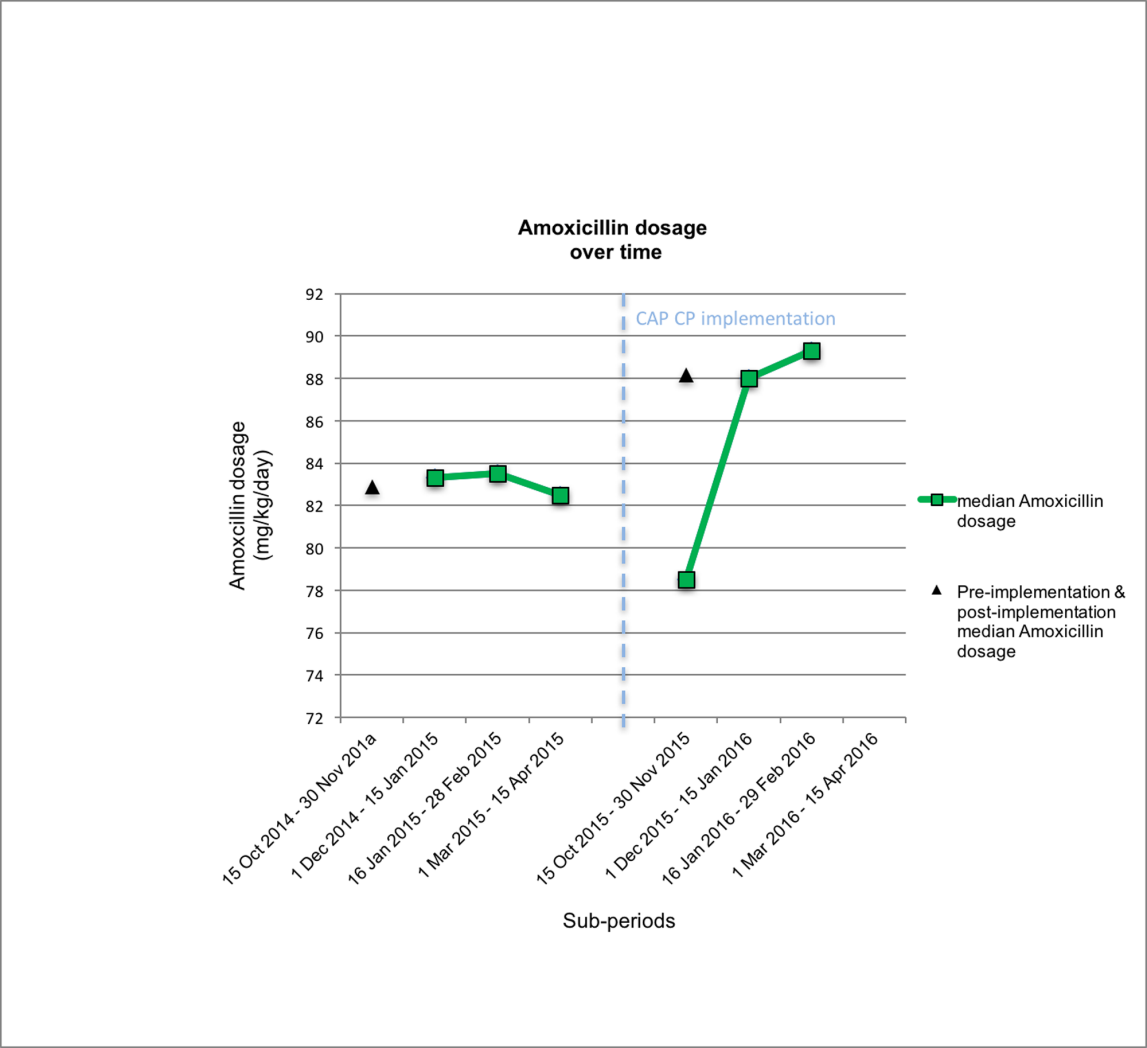
Median amoxicillin dosage over time.

The most commonly prescribed antibiotic for outpatients with CAP was amoxicillin. Pre-intervention median dosage corresponds to 82.9mg/kg/day (range 28.6–102). Post-intervention median dosage was 88.15mg/kg/day (range 64–95.5) (p = 0.03) as recommended in the CP (Fig 4).

### Antibiotic prescription in inpatients

#### Changes in prevalence of antibiotic prescriptions for CAP in inpatients

We observed an increase in narrow-spectrum regimens prescribed in the post-CP period: 6/18 children (33.3%) after CP implementation received exclusively narrow-spectrum antibiotics in contrast with 0% in the pre-intervention period (p = 0.02). Broad-spectrum antibiotics also showed a decreasing trend (14/14 pre-CP, 12/18 post-CP).

Median DOT for the pre-intervention period was 18.5 days (range 11–32), for the post-intervention it was 10 (range 3–26) (p = 0.004).

We reported a statistically significant increase in use of ampicillin and amoxicillin and a concomitant decrease in use of cephalosporins and macrolides. Furthermore, use of amoxicillin-clavulanate, ampicillin-sulbactam, carbapenems and glycopeptides was abandoned. Clindamycin was prescribed only in the post-CP period.

#### Changes in prevalence of broad-spectrum antibiotic prescriptions for CAP

Broad-spectrum antibiotic use was assessed through median bsDOTs: pre-intervention median bsDOT was 17 (range 11–24) and it decreased to 4.5 (range 1–23) in the post-intervention period (p<0.0001).

Broad-spectrum in relation to overall antibiotic use evaluated through bsDOT/DOT ratio decreased from 0.83 in the pre-CP to 0.41 in the post-CP period (p<0.0001).

#### Changes in duration of therapy and length of stay

Pre-CP median LOT was 11 days (range 5–17), while post-CP LOT was 10 days (range 3–15) (p = 0.06). Pre-intervention ratio of DOT/LOT, which measures the quantity of antibiotics prescribed per day was 1.70, while post-intervention DOT/LOT was 1.26.

Median LOS in the pre-CP period was 5 days (range 3–16) and in the post-CP period, 4 days (range 2–14) (p = 0.23).

#### Changes in dosage for the most commonly prescribed antibiotics for CAP

The CAP CP developed for this study recommends ampicillin dosage of 200–300 mg/kg/day. In the pre-CP period, there were no ampicillin prescriptions. In the post-intervention period, 6/18 (16.7%) patients received ampicillin with median dosage of 200 mg/kg/day (range 200–307.7).

The recommended dosage of ceftriaxone in our CAP CP is 50–100 mg/kg/day. Ceftriaxone was prescribed in 8/14 (57.1%) pre-CP patients and 8/18 (44.4%) post-CP. The median dosage was 75 mg/kg/day both pre-intervention (range 38.5–100) and post-intervention (range 75–100).

### Treatment failure in outpatients

As a balancing measure, we assessed treatment failure: 44/56 (78.5%) children were available for CAP follow-up in the baseline period, in comparison with 37/41 (90.3%) in post-intervention period.

The two groups were compared for prevalence of prescriptions: amoxicillin and broad spectrum antibiotic prescriptions did not show significant differences before and after CP implementation, though trends for all antibiotics indicated improvement post-intervention (Table 3).

**Table 3:** Antibiotics prescriptions for outpatients follow-up.

	**Pre-intervention period**		**Post-intervention period**		**p value**
**Number of outpatients available for follow-up**	44		34		
**Number of prescriptions**	56		39		
**Prescriptions/patients ratio**	1.3		1.1		
	N	%	N	%	
Amoxicillin	28	50	25	64.1	0.17
Cephalosporins	9	16.1	4	10.3	0.42
Macrolides	10	17.8	3	7.7	0.16
Amoxicillin-clavulanate	9	16.1	7	17.9	0.97

In the pre-CP period, treatment failure occurred in 2.3% (1/44) of cases, while 11.8% (4/34) failed treatment in the post-CP period (p = 0.29). All these cases consist of change of antibiotic for persistence or worsening of symptoms.

### Treatment failure in inpatients

Twelve out of 14 (85.7%) parents were available for a follow-up call in the pre-intervention period and 13/18 (72.2%) in the post-intervention period (p = 0.36). In the pre-CP period 83.3% (10/12) of prescribed therapy was effective, with 2/12 cases of antibiotic change during hospitalization for persistence or worsening of symptoms. There was no significant change in the post-CP period, where 84.6% (11/13) of prescribed therapies was effective and for 2/13 patients’ antibiotics were changed for persistence of symptoms.

## Discussion

In accordance with the literature, the highest prevalence of CAP was observed in children <5 years of age in our population [22].

Current recommendations, reflected in our CAP CP, indicate that children with a clinical diagnosis of pneumonia should receive antibiotics, as bacterial and viral pneumonia cannot be reliably distinguished from each other [8]. Narrow-spectrum monotherapy (amoxicillin) is the first option for mild CAP in fully immunized children, as *S*. *pneumoniae* accounts for 21–44% of disease [23–28].

Use of macrolides is only appropriate if atypical bacterial ethology is suspected, as the use of azithromycin has been associated with the selection of resistant organisms because of its prolonged serum elimination half–life [1,8].

According to other authors, our study showed relevant changes in physicians’ prescribing behaviours for both outpatients and inpatients [16,17]. In contrast to other settings, where significant effects were achieved only during the second year of the intervention, in this case changes took place immediately after CP implementation [11].

During the post-intervention period more narrow-spectrum regimens were prescribed for fewer days.

In our population, macrolide over-prescription in the pre-CP period may have resulted from the perception that two antimicrobials were more reliable, as only one patient was actually infected with *Mycoplasma pneumoniae* in this period. Indeed, the pre and post populations were similar in terms of number of tests performed to detect *M*. *pneumoniae* infection and positive tests (only one in the pre-CP period).

All inpatients were admitted to the PACU for an episode of moderate CAP.

No statistical difference was reported between the two groups before and after implementation with regard to sex, age, symptoms, *M*. *pneumoniae* IgM serology positivity and presence of complications (hypoxia, pleural effusion, necrotizing pneumonia).

After CAP CP implementation, prescription of narrow-spectrum regimens (ampicillin or amoxicillin) among inpatients decreased significantly, with a concomitant decrease of broad-spectrum antibiotics. This is in line with most recent recommendations, which suggest high doses of narrow-spectrum β-lactams as the first-line parenteral therapy for moderate CAP if the child is fully immunized or is not admitted for a previous amoxicillin treatment failure. Alternatively, parenteral third generation cephalosporins are recommended [1] when these criteria are not met.

Median DOTs showed a substantial decrease from 18.5 to 10 days in pre- versus post-CP periods, indicating a decrease in the prevalence of antibiotic prescriptions. As reported by Smith et al [17], we saw a significant increase in use of ampicillin and amoxicillin and decrease in cephalosporins and macrolides. This is an important achievement for inpatients: before the CP implementation physicians didn’t even consider ampicillin for moderate CAP treatment in our centre.

Furthermore, use of broad-spectrum antibiotics like amoxicillin-clavulanate, ampicillin-sulbactam, carbapenems and glycopeptides were abandoned after CP implementation in our centre, while clindamycin was prescribed in the post-CP period only in case of complicated pneumonia (parapneumonic effusion, necrotizing pneumonia).

Carbapenems are one of the β-lactams with the broadest antibacterial spectrum currently available, with a relatively low rate of adverse effects. They are recommended as “last-line agents” for severe infections or resistant bacteria, since carbapenems are not destroyed by most β-lactamases [29]. From this study, it emerged that their empiric use was not exclusively for severe nosocomial infections in critically ill patients, but was prescribed as drug of choice for moderate CAP during pre-intervention period, as also reported by other authors [30]. The wide use of these lifesaving drugs is problematic due to the emergence of carbapenem-resistant bacteria which cause severe infections [31–35].

The dramatic change in antimicrobial choices is attributable to the shift in suggested first-line therapy for CAP and, since the starting therapy is established for both outpatients and inpatients by the PED, improvements in PED prescriptions determine improvements in PACU prescriptions, even because the two wards share the same medical staff.

Indeed, starting with penicillin (amoxicillin/ampicillin) gives physicians the possibility to observe children for 48–96 hours and, in case of persistence of symptoms, to switch to a third-generation cephalosporin. On the other hand, physicians starting with ceftriaxone are more prone to change to a broader spectrum antibiotic such as carbapenem.

Furthermore, the introduction of clindamycin recommendations for complicated moderate CAP gave the opportunity to avoid glycopeptides, hence reducing their use.

DOT, LOT and DOT/LOT ratio analysis was performed to describe inpatient prescriptions. Our intervention resulted in a statistically significant decrease in overall median DOT (narrow and broad-spectrum) from 18.5 to 10, as well as bsDOT from 17 to 4.5. LOT median did not significant decrease, despite CAP guidelines recommending 7 days of therapy in case of uncomplicated CAP and 14 days if complicated (parapneumonic effusion, necrotizing pneumonia). This suggests pediatricians are more inclined to change their attitude towards the choice of antibiotic prescribed rather than the duration of therapy.

The increased use of ampicillin/amoxicillin also resulted in a decrease of the median LOS after CP implementation. Indeed, a rapid and uneventful improvement during the first 24–48 hours after ampicillin administration has a favourable impact on switch to oral antibiotic and early discharge [36].

For both outpatient and inpatient populations, no differences in treatment failure were reported despite a remarkable decrease in broad-spectrum antibiotic prescription. During the post-CP period, an increase in treatment failure was reported in outpatients but it was not statistically significant. This may be due to relatively low sample sizes and to the very low occurrence of treatment failure overall, even in the pre-CP period. Continuing surveillance is needed to confirm this trend, as such a substantial reduction in treatment failure after CP would be clinically significant.

This study has strengths and limitations. It is the first study that evaluates the effectiveness of ASP through CPs in an Italian hospital. This intervention was designed to be feasible and was developed by a multidisciplinary team to guarantee a high quality and level of coordination, with cooperation between the Infectious Diseases and PED teams. Furthermore, following CP presentation a prominent educational campaign included lectures and distribution of handy pocket cards and posters.

This is the first study with a phone call follow-up to assess antimicrobial stewardship in the PED context, allowing evaluation of antibiotic changes for persistence of symptoms or side effects.

Limitations include the retrospective nature of the analysis, its single-centre setting, the short period of observation,the inability to assess appropriateness of single antimicrobial prescriptions and the small amount of inpatients.

Thus, a longer term follow up study evaluating the longevity of observed changes in antimicrobial prescription is warranted to analyse further improvements, as well as expanding to include other Italian PED for validation of this tools.

## Conclusions

This study provides evidence that clinical pathway implementation in an Italian PED setting is an effective tool for antimicrobial stewardship, appearing to be associated with the kind of treatment children receive.

An evidence-based CP supplemented by educational and explanatory lectures was associated with significant changes in prescribing habits of physicians at our centre, decreasing the use of broad-spectrum antibiotics in favour of narrow-spectrum, and reducing the length of therapy without increasing treatment failure both for outpatients and inpatients.

## Supporting information

S1 Fig Clinical pathway. (PDF)

## Abbreviations

ASPs: Antimicrobial stewardship programs
bsDOT: Broad-spectrum days of therapy
CP: Clinical Pathway
CAP: Community-acquired pneumonia
DOT: Days of therapy
LOS: Length of hospital stay
LOT: Length of therapy
PACU: Pediatric Acute Care Unit
PED: Pediatric Emergency Department
