# Supplementary material for: Effects of clinical pathway implementation on antibiotic prescriptions for pediatric community-acquired pneumonia
Source: PLoS One. 2018 Feb 28;13(2):e0193581. doi: 10.1371/journal.pone.0193581 (PMC5831636; doi:10.1371/journal.pone.0193581)
Supplement: S1 Fig — (PDF) [file pone.0193581.s001.pdf]

Community-Acquired Pneumonia Pocket Guide

Children > 90 days

Exclusion criteria:
Chronic lung disease (except asthma)
Immunodeficiency, HIV
Tracheostomy
Risk factors for aspiration pneumonia
IV antibiotic therapy during previous 30 d
HA pneumonia (<14 d from previous hosp)

OUTPATIENT

Mild Pneumonia
- -Age > 3 months
- -Absence of respiratory distress
- -SatO2 > 92% in room air
- -Non-toxic appearance
- -Ability to tolerate oral medications and fluids
- -Adequate observation/follow-up care

Consider Chest X-ray only if diagnosis uncertain. Routine CXRs are not necessary to confirm the CAP diagnosis

Diagnosis of bacterial CAP supported?
- Yes → Fully Immunized?**
  - Yes → **First line initial therapy:** **Amoxicllin** 90 mg/kg/day, div Q8hrs; (max: 1g/dose) X 7 d. **Alternatives if PCN allergy*****
    - → No clinical improvement > 48-h. Reassess the patient → Amoxicillin-clav (see below); → Ceftriaxone (inpatient first line therapy); → No clinical improvement > 48 h (PICU)
  - No → **Amoxicillin-clav** 90/6.4 mg/kg/day, div Q8hrs max: 1g/dose x 7 d. OR think about ***M. pneumoniae*** infection*
- No → Consider alternative etiologies (viral etiology common in children <5 y) !

INPATIENT

Moderate Pneumonia
- -Age < 3 months
- -Moderate-severe dyspnea
- - SatO2 < 92% in room air
- -Alteration in mental status
- -Concern for inadequate outpatient care/FU
- -Dehydration, vomiting, inability to take oral meds
- -Clinical concern for inpatient-level observation

INPATIENT PICU

Severe Pneumonia
- -Need for mechanical ventilator support with artificial airway
- -New or increased CPAP or BiPap support
- -Severe resp distress or concern for respi failure
- -Hypoxemia (SatO2 < 90%) despite significant O2 (40% high flow nasal cannula, 100% non-rebreather mask)
- -Systemic signs of inadequate perfusion
- -Parapneumonic effusion requiring emergent drainage

Recommended: Chest X-ray, CBC w/diff, CRP
Sometimes recommended: Blood cx, Respiratory viral panel, Mycoplasma rapid serology*, Sputum cx (in children that can produce a sputum sample)

Diagnosis of bacterial CAP supported?
- No → Consider alternative etiologies
- Yes → Effusion on CXR?
  - No → Fully Immunized?**
    - Yes → **First line initial therapy:** Ampicillin 200-300 mg/kg/day div Q6hrs max: 2g/dose x 7 d. **Alternatives if PCN allergy*****. **Switch from IV to PO and discharge** when improving within 48-72 h based upon: Resp rate, resp effort, fever, O2 requirement. **Duration**: 7 d or at least 48 h from resolution of fever and tachypnea (whichever is longer), including PO/outpatient therapy
      - → No clinical improvement > 48 h. Reassess the patient → Complicated Pneumonia; → Ceftriaxone
    - No → **First line initial therapy:** Ceftriaxone 50–100 mg/kg/day Q24hrs or div Q12hrs or cefotaxime 150 mg/kg/day div Q8hrs. **Switch from IV to PO and discharge** when improving within 48-72 h based upon: Resp rate, resp effort, fever, O2 requirement. **Duration**: 7 d, or at least 48 h from resolution of fever and tachypnea (whichever is longer), including PO/outpatient therapy
      - → No clinical improvement > 48 h. Reassess the patient → Severe Pneumonia
  - Yes → Complicated Pneumonia

Complicated Pneumonia
(moderate parapneumonic effusion)
Clindamycin 40 mg/kg/day div Q8hrs; max 2.7 g/day
AND
Ceftriaxone 100 mg/kg/day Q24hrs; max: 2 g/dose
Alternatives if PCN allergy:
Instead of ceftriaxone
Levofloxacin (IV/PO) ≥ 6mo and < 5 yrs:20 mg/kg/day div Q12hrs ≥ 5yr: 10 mg/kg/day Q24hrs; max: 500 mg
OBTAIN US OF CHEST AND CONSULT GENERAL SURGERY FOR EFFUSIONS REQUIRING DRAINAGE****
Duration: 7 d from drainage.
If not amenable to drainage, 7 d from afebrile

Severe Pneumonia
(including complicated severe pneumonia)
Vancomycin 60 mg/kg/day (max 500 mg/dose), div Q6hrs
AND
Ceftriaxone 100 mg/kg/day Q24hrs max: 2 g/dose
Alternatives if PCN allergy:
Vancomycin 60 mg/kg/day (max 500 mg/dose) div Q6hrs
AND
Levofloxacin (IV/PO) ≥ 6mo and < 5 yrs:20 mg/kg/day div Q12hrs ≥ 5yr: 10 mg/kg/day Q24hrs; max: 500 mg
IV-PO Switch and Discharge: Chest tube out, Feeding well, No O2 requirement, Fever curve trending down, able to take PO abx

No clinical improvement > 48 h. Reassess the patient

---

*Confirmed/Presumed ***M. pneumoniae*** infection: **Azithromycin**: 10 mg/kg on day 1, single dose (max: 500 mg/day), followed by 5 mg/kg/day, once daily, days 2-5 (max: 250 mg/day) **OR Clarithromycin** 15 mg/kg/day div Q12hrs for 10 days
**Not fully immunized for H. influenzae type b and S. pneumoniae (less than 2 doses)
*** **Alternatives if PCN allergy: If not IgE mediated**: 2 or 3 generation oral cephalosporin, only for mild pneumonia!! (**cefuroxime** (30 mg/kg/day div Q12hrs), **cefpodoxime**, (10 mg/kg/day div Q12hrs) for 7 d, **If IgE mediated: Clindamycin** (IV) 40 mg/kg/day div Q8hrs; max 2.7 g/day or (PO) 30 mg/kg/day div Q8hrs; max 1.8 g/day, **Levofloxacin**: (IV/PO) ≥ 6mo and < 5 yrs: 20 mg/kg/day, div Q12hrs ≥ 5yr: 10 mg/kg/day, Q24hrs max: 500 mg
****Large and/or growing in size, Impairing pulmonary function, Organized and loculated

References:
The Management of Community-Acquired Pneumonia in Infants and Children Older Than 3 Months of Age: Clinical Practice Guidelines by the Pediatric Infectious Diseases Society and the Infectious Diseases Society of America
Harris M et al, British Thoracic Society guidelines for the management of community acquired pneumonia in children: update 2011. Thorax
